# LGC-DBP: the method of DNA-binding protein identification based on PSSM and deep learning

**DOI:** 10.3389/fgene.2024.1411847

**Published:** 2024-06-05

**Authors:** Yiqi Zhu, Ailun Sun

**Affiliations:** Department of Computer Science and Technology, College of Computer and Control Engineering, Northeast Forestry University, Harbin, China

**Keywords:** bioinformatics, deep learning, position-specific scoring matrix, inception convolution, channel attention mechanism

## Abstract

The recognition of DNA Binding Proteins (DBPs) plays a crucial role in understanding biological functions such as replication, transcription, and repair. Although current sequence-based methods have shown some effectiveness, they often fail to fully utilize the potential of deep learning in capturing complex patterns. This study introduces a novel model, LGC-DBP, which integrates Long Short-Term Memory (LSTM), Gated Inception Convolution, and Improved Channel Attention mechanisms to enhance the prediction of DBPs. Initially, the model transforms protein sequences into Position Specific Scoring Matrices (PSSM), then processed through our deep learning framework. Within this framework, Gated Inception Convolution merges the concepts of gating units with the advantages of Graph Convolutional Network (GCN) and Dilated Convolution, significantly surpassing traditional convolution methods. The Improved Channel Attention mechanism substantially enhances the model’s responsiveness and accuracy by shifting from a single input to three inputs and integrating three sigmoid functions along with an additional layer output. These innovative combinations have significantly improved model performance, enabling LGC-DBP to recognize and interpret the complex relationships within DBP features more accurately. The evaluation results show that LGC-DBP achieves an accuracy of 88.26% and a Matthews correlation coefficient of 0.701, both surpassing existing methods. These achievements demonstrate the model’s strong capability in integrating and analyzing multi-dimensional data and mark a significant advancement over traditional methods by capturing deeper, nonlinear interactions within the data.

## 1 Introduction

The interaction between DNA and proteins plays an indispensable role in many biological processes such as DNA replication Kornberg (2000), Jones et al. (1987), transcription Sobell (1985), and repair Wood et al. (2001). Understanding the underlying mechanisms of DNA-protein interactions can help elucidate these biological processes.DNA Binding Proteins (DBPs) are crucial components in steroids, antibiotics, anticancer drugs, and treatments for genetic diseases. Accurately identifying DBPs is a critical step in understanding their interactions. In the early stages, researchers inferred the presence of DBPs through wet lab experiments such as microarray chromatin immunoprecipitation (ChIP-chip), genetic analysis Buck and Lieb (2004), and X-ray crystallography Chou et al. (2003), all of which have achieved considerable success. However, these efforts depend on their sources of features: methods based on three-dimensional (3D) structures and sequence-driven methods. The performance of structure-based methods is considered accurate in identifying and characterizing protein activity from 3D structures. The precise identification of protein 3D structures still relies on expensive and time-consuming wet lab experiments, often resulting in unclear 3D structural information for many proteins. Although the field of protein structure prediction has made significant advances with methods such as I-TASSER Yang and Zhang (2015), AlphaFold Senior et al. (2020), and AlphaFold2 Rahman et al. (2018), the accuracy of these protein structure predictions cannot be guaranteed.

Sequence-based methods proposed and implemented over the past few decades consistently frame DBP prediction as a binary classification task. The classification of DNA-binding proteins involves various techniques and strategies. Traditional methods include models based on machine learning and feature engineering, such as the Support Vector Machine (SVM), which trains on features derived from sequence, structure, and functional domains. In recent years, the development of deep learning has introduced new recognition models such as Convolutional Neural Networks (CNNs), Recurrent Neural Networks (RNNs), and models incorporating attention mechanisms. For example, Liu et al. (2015a). Developed an iDNAPro-PseAAC method, which constructs a predictive model for DNA-binding proteins by combining SVM Lin et al. (2011). Integrated amino acid distance-pair coupling information and simplified amino acid alphabet profiles into a general pseudo amino acid composition (PseAAC) vector, establishing a novel predictor named “iDNA-Prot—dis.” Wei et al. (2017). Proposed a novel method for identifying DNA binding proteins to establish a new predictor called Local-DPP by combining local Pse-PSSM features with a random forest classifier. Liu et al. (2015b). They proposed PseDNA-Pro, which creates a vector of characteristics composed of three sequence-based features, including the overall amino acid composition, the pseudo amino acid composition (PseAAC) proposed by Zhou et al., and the physicochemical distance transformation. These features consider the sequence composition of proteins and integrate the sequence-order information of amino acids in proteins. These feature vectors were input into an SVM for DNA-binding protein identification. Hu et al. (2019), Hu et al. (2021). Also, the TargetDBP and TargetDBP + methods made feature extraction and selection innovations Zhang et al. (2021). A DBPs prediction method was introduced based on a stacked ensemble classifier, StackPDB. Firstly, protein sequence features were extracted using pseudo amino acid composition (PseAAC), pseudo-position-specific scoring matrix (PsePSSM), position-specific scoring matrix-transition probability composition (PSSM-TPC), evolutionary distance transformation (EDT), and residue probing transformation (RPT). Secondly, extreme gradient boosting-recursive feature elimination (XGB-RFE) was employed to obtain an excellent feature subset. Finally, the best features were applied to construct StackPDB using a stacked ensemble classifier composed of XGBoost, LightGBM, and an SVM. The MsDBP predictor proposed by Du et al. (2019). Employs a deep neural network framework incorporating multi-scale sequence features. The DBPboost method proposed by Sun et al. (2024). Using eight feature extraction methods, improve the feature selection step by initially selecting some features and performing feature selection after feature fusion. Furthermore, it optimizes the application of the differential evolution algorithm in feature fusion to enhance its performance.

Although many effective DNA-binding protein identification methods have been developed, their performance has room for improvement. Firstly, although machine learning-based methods for classifying DNA-binding proteins, such as TargetDBP+, offer good interpretability, their performance is still not as good as deep learning frameworks. Secondly, while models based on deep learning frameworks, such as MSDBP and AlphaFold, have been successful in protein prediction, they also have their respective shortcomings: the MSDBP model is not complex enough to effectively capture the characteristics of DNA-binding proteins. Although AlphaFold excels in structural prediction, it is primarily designed for structure prediction rather than classification or functional identification. Therefore, it has yet to be optimized to enhance the performance in recognizing DBPs. This indicates that developing a high-accuracy deep learning prediction model tailored explicitly for DBPs is feasible and necessary.This study introduces a DNA-binding protein recognition method named LGC-DBP, which aims to enhance recognition performance by combining various neural network architectures. Long Short-Term Memory (LSTM) initially captures long-term dependencies and sequence features within protein sequences. The output of the LSTM is then fed into a module comprising a Graph Convolutional Network (GCN) and dilated convolution layers, whose outputs are weighted and summed using a sigmoid function. These layers extract features at different scales and provide contextual information fused through weighted summation to enhance model performance further. Subsequently, the weighted summation result is passed through an improved channel attention module to strengthen critical features in the protein sequence. This channel attention mechanism automatically learns and highlights significant features, enhancing model performance and robustness. It employs three inputs and calculates weights through a fully connected layer. This method seamlessly integrates multiple neural network structures, leveraging their feature extraction and sequence modeling advantages to improve the accuracy and generalization ability in DNA-binding protein recognition. Through experimental validation, this method has achieved significant performance improvements in protein identification tasks, achieving a prediction accuracy of 88.01% and a Matthews correlation coefficient of 0.738, significantly higher than most existing state-of-the-art DBP prediction methods. This provides new insights and approaches for further research and application in DNA-binding proteins.

The remainder of this study is as follows: The second section describes the structure of the method used in this paper. In the third section, we provide details of the datasets used in the experiments and investigate, compare, and interpret the experimental results. Finally, in the fourth section, we discuss and summarize our work.

## 2 Propose method

### 2.1 Benchmark data sets

To assess LGC-DBP’s performance, we utilized the UniSwiss dataset, previously used in our group’s previous investigation. This dataset encompasses proteins from various species, such as humans, mice, and *Arabidopsis thaliana* (A. thaliana), comprising 9,762 proteins from the UniProtKB/Swiss-Prot database. Each protein within the dataset contains more than 50 residues, and the sequence identity between any two DBPs (or non-DBPs) is less than 25%. The UniSwiss dataset is partitioned into two subsets: UniSwiss-Tr, serving as the training set, which comprises 4,500 DBPs and an equal number of non-DBPs, and UniSwiss-Tst, acting as the independent test set, consisting of 381 DBPs and 381 non-DBPs. Access to the UniSwiss dataset is provided at https://github.com/jun-csbio/TargetDBPplus/.

### 2.2 Data preprocessing

The benchmark dataset comprises FASTA sequences, which require their conversion into PSSM(Position Specific Scoring Matrices) Jones (1999), Jeong et al. (2010) for subsequent analysis. PSSM is a commonly used matrix representation method in bioinformatics, reflecting the conservation of each amino acid at specific positions within a set of sequences. Using data from multiple sequence alignment (MSA), PSSM provides valuable information on proteins’ evolutionary relationships and functional features. Each row of the PSSM matrix corresponds to an amino acid residue, and each column is placed in a specific position in the protein sequence. The elements in the matrix represent the logarithmic odds scores of each amino acid observed at a given position based on the frequencies observed in the sequence alignment. PSSM is typically used as input features for various bioinformatics tasks, including protein structure prediction, function prediction, and protein-ligand binding site prediction.

Many protein classification methods based on PSSM often perform additional feature extraction on top of the PSSM, overlooking the inherent feature information contained within the PSSM itself. Therefore, in this study, the information directly extracted from the PSSM matrix is selected as the input tensor and then fed into the model for training.

### 2.3 Structure of LGC-DBP

Our model adopts a fully supervised approach to address the issue of data scarcity by introducing an ensemble or stack of multiple models. The fundamental idea behind model ensembling is to combine several different models, each tasked with handling different aspects of the problem or providing diverse feature representations. By synthesizing the predictions of multiple models, we can significantly enhance overall performance, which helps overcome challenges associated with small data volumes and boosts the model’s generalization capability. In the practical implementation of this study, we initially generate a PSSM using the NCBI BLAST tool from a given DNA-binding protein sequence. The PSSM is derived from multiple sequence alignments and accurately reflects the probability of occurrence of different amino acids at each position. The data set for this study includes a training set composed of 4,500 DNA-binding and 4,500 non-DNA-binding protein sequences, along with an independent test set containing 381 DNA-binding and 381 non-DNA-binding protein sequences. Each protein sequence is associated with a PSSM to capture the frequency and conservation of amino acids at each position.

Given that multiple PSSM matrices increase the dimensionality and complexity of the data, we have applied dimensionality reduction techniques to the PSSM matrices before feeding them into an LSTM network. The dimensionality reduction utilizes ensemble-based feature selection and Principal Component Analysis (PCA). The LSTM network is particularly suited for processing sequential data, as it can capture long-term dependencies and extract time or structure-related features from the sequence. The output from the LSTM is then passed through an enhanced Gated Inception Convolution module. This module combines the advantages of graph convolutional networks and dilated convolutional networks, employing gating mechanisms and dilated convolutions to capture and integrate deep features of DNA-binding proteins efficiently. The output undergoes further processing by an improved channel attention module. This module modifies traditional channel attention to increase flexibility, allowing it to learn valuable positional information from different channels of the PSSM. The application of these technologies makes the model more efficient and precise in handling complex biological sequence data.

Finally, the features are transformed into prediction probabilities through a fully connected layer, employing the softmax function to distinguish between DBPs and non-DNA-binding proteins (Non-DBPs). The model is trained on the UniSwiss-Tr dataset, optimizing a loss function based on cross-entropy to predict and classify DNA-binding proteins. This integrative approach, which utilizes multiple modules and techniques, provides an efficient and accurate solution for precisely predicting protein functions. This study selects cross-entropy loss as the loss function for the model, computed as shown in Formula 3:

$$LOSS=-\overset{20}{\underset{k=1}{\sum }}y_{i}log\left({\hat{y}}_{i}\right)$$ (1)

Here *y*_*i*_ and ${\hat{y}}_{i}$ denote the actual label and the predicted probability value, respectively. Ultimately, the Adam algorithm optimizes this loss function Kingma and Ba (2014). For implementation, we use Facebook’s PyTorch library, an open-source tool Paszke et al. (2019).

#### 2.3.1 Long short-term memory (LSTM)

LSTM is a recurrent neural network (RNN) architecture designed to overcome the vanishing gradient problem and capture long-term dependencies in sequential data. Hochreiter and Schmidhuber introduced it in 1997.

At its core, an LSTM consists of memory cells that maintain an internal state, allowing them to retain information over time. Three gates control these cells: the input gate (i), the forget gate (f), and the output gate (o). Each gate regulates the flow of information into and out of the cell. In addition, the LSTM incorporates a cell state (C) and a hidden state (h), both of which are updated at each time step.

PSSM is derived from multiple sequence alignment (MSA), reflecting the conservation of each amino acid at specific positions in the protein sequence. Due to the long-term dependencies in protein sequences, LSTM is well-suited for capturing these dependencies in sequence data, thus better modeling the features within PSSM. In traditional RNNs, the gradients may vanish or explode as time steps increase during backpropagation. However, LSTM, with its gated structures such as input gates, forget gates, and output gates, effectively controls the flow of gradients, thereby avoiding the vanishing gradient problem and more effectively capturing long-term dependencies in sequence data. Additionally, LSTM can learn and extract abstract features from PSSM, facilitating information interaction across multiple time steps and effectively capturing semantic and syntactic information in sequence data, thereby improving classification performance.

#### 2.3.2 Gated inception convolution

This module is an improvement based on Inception Convolution. Inception Convolution, also known as GoogleNet Inception, is a convolutional neural network (CNN) Fukushima (1980) architecture introduced by Google Szegedy et al. (2015). It employs multiple parallel convolutional layers with different filter sizes to efficiently capture features at various spatial scales, thereby avoiding gradient vanishing. The network can extract features using parallel convolutional operations while minimizing computational costs. Inception Convolution has been widely used in image classification and recognition tasks due to its ability to balance computational efficiency and model performance.

The improvements to Inception Convolution in this study are divided into two aspects: replacing the convolutional layers and using a sigmoid function for weighting.

First, this study replaces the convolutional layers of this module with GCN Kipf and Welling (2016) and dilated convolution. The advantage of replacing the two convolutional layers in Inception Convolution with GCN and dilated convolution lies in the comprehensive utilization of the strengths of different convolutional layers. GCN effectively captures both local and global information of graph-structured data and is particularly suitable for processing such data (Wang et al., 2023a), including protein sequences. On the other hand, dilated convolution extracts feature at different receptive fields, increasing the network’s sensitivity to features. This combination enables a more comprehensive capture of spatial features and relationships in the input data, enhancing the model’s representation ability and classification performance on complex data. Moreover, GCN and dilated convolution can reduce model complexity, accelerate training speed, and improve efficiency and performance.

The graph convolutional layer can be represented as a non-linear function, as shown in Formula 4:

$$H^{l+1}=\sigma \left(AH^{l}W^{l}\right)$$ (2)

Where *W* ^*l*^ is the weight parameter matrix for layer *l*. *σ* is the non-linear activation function, with ReLU utilized in this study. *H* ^*l*^ represents the input values for this layer, while *H* ^*l*+1^ represents the input values for the next layer.

Dilated Convolution Yu et al. (2017), also known as atrous convolution or convolution with holes, injects holes into the standard convolutional kernel to expand the model’s receptive field. In contrast to regular convolution operations, dilated convolution introduces an additional parameter: the dilation rate, which refers to the number of intervals between the points of the convolution kernel. For instance, the dilation rate is set to 1 in standard convolution operations.

**FIGURE 1:**
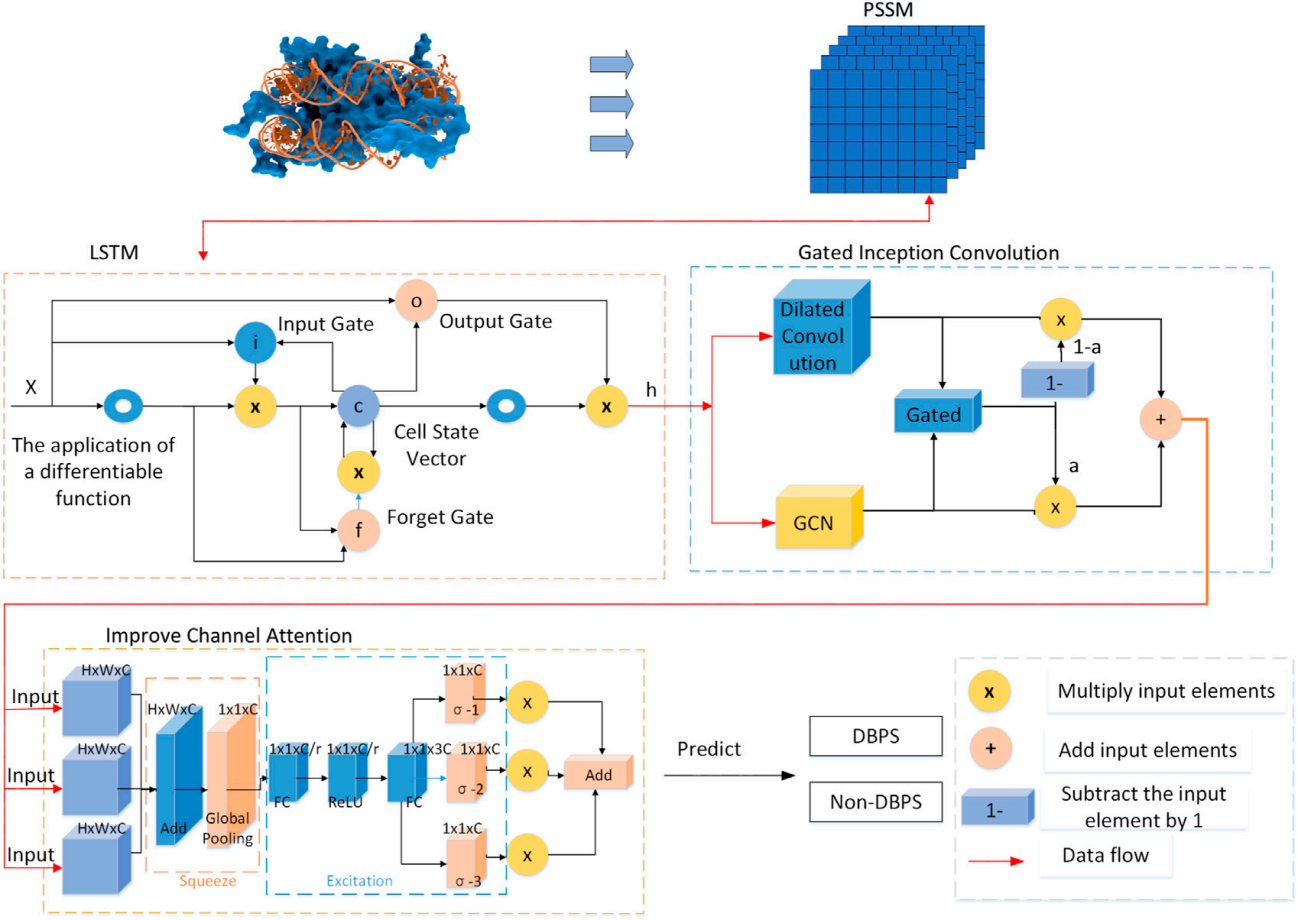
Architecture of LGC-DBP.

**FIGURE 2:**
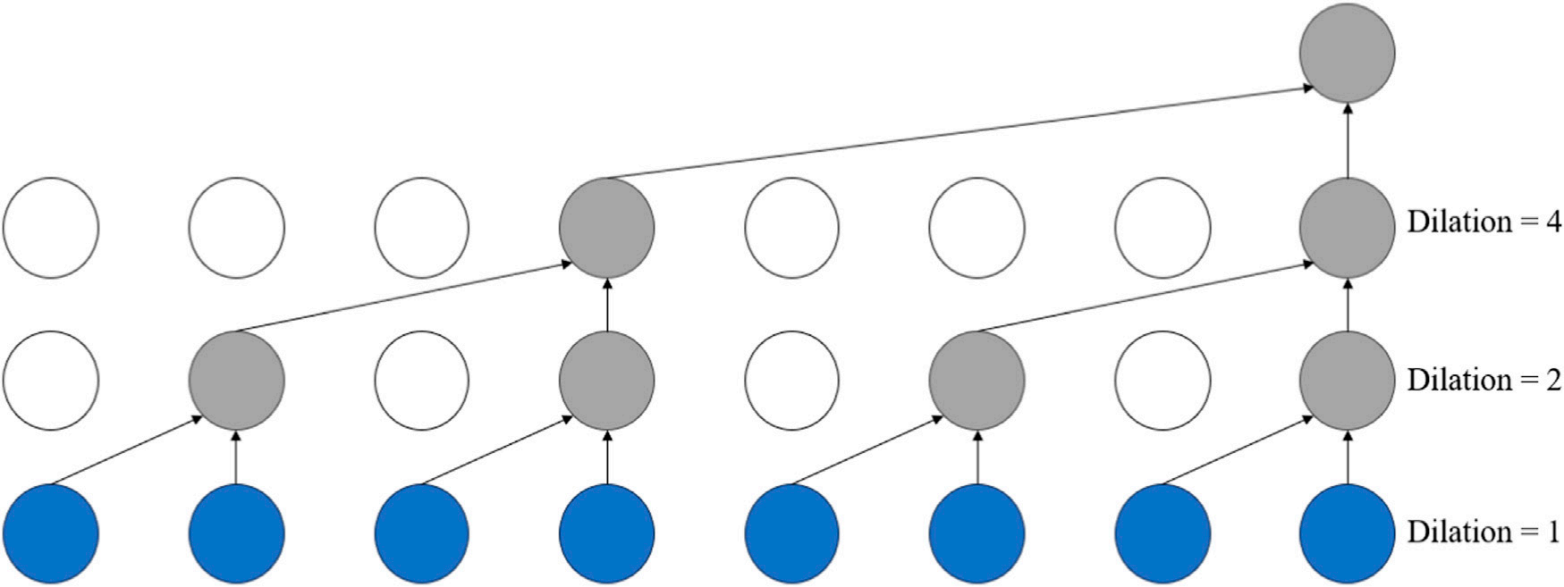
The structure of the dilated convolutional network.

The dilated convolution layers in this module can extract features at different scales, enabling the capture of spatial structures and contextual information of various sizes. This capability helps to represent features within protein sequences more accurately. The structure diagram of the dilated convolutional network is shown in Figure 2. Introducing dilated convolution and GCN into the Inception Convolution module can improve overall performance, but we aim to enhance DNA-binding protein recognition further. Therefore, we introduce a gate fusion mechanism here, adding a gating unit to the Inception Convolution to receive the output of the GCN and the Dilated Convolution and to generate a weight a, computed by the sigmoid function. Multiplying “a” with the output of GCN and “1-a” with the output of dilated convolution achieves the weighting of the two, enhancing their connection. Finally, the weighted results are summed to serve as the overall output of this module.

After the improvements to Inception Convolution described above, we incorporated the advantages of GCN and dilated convolution into Inception Convolution and introduced the weighting method of gated units. We refer to the improved Inception Convolution as Gated Inception Convolution.

#### 2.3.3 Improved channel attention

The channel attention mechanism has several advantages in deep learning Vaswani et al. (2017), Hu et al. (2018). Firstly, it dynamically adjusts attention to different channels, enabling the model to learn and utilize essential features within the input data effectively. Secondly, channel attention can adaptively learn the weights of each channel, making the model more flexible and adaptable to different input data and tasks. Additionally, channel attention helps improve the model’s generalization ability, reducing the risk of overfitting and enhancing performance and robustness. In general, the channel attention mechanism provides an effective way for deep learning models to improve their understanding and representation of input data.

However, the original channel attention mechanism has some limitations. First, it cannot adapt to different tasks and datasets because it uses a fixed attention mechanism across all channels. Second, it may be difficult to effectively capture and utilize spatial and contextual information in the data. Furthermore, the original mechanism may need to fully address the variations in the importance of different channels in different contexts. Therefore, there is a need for improvement to make the attention mechanism more flexible, adaptable, and capable of capturing complex patterns in the data, thereby enhancing the model’s performance and generalization ability across various tasks and datasets.

First, we modified the input of the channel attention by employing three different convolutional layers to obtain information at three different scales, allowing for better extraction of information from other dimensions of the input tensor. In the squeeze stage, we added them to fuse multi-scale information and then compressed it through global pooling. In the excitation stage, there are two fully connected layers. Initially, in the first fully connected layer, we reduced the number of channels, and then in the second fully connected layer, we restored the number of channels to 3C, facilitating the subsequent weighting of the three inputs. Then, we segmented them into three parts along the channel dimension, applied sigmoid functions to weight the three inputs separately, and added the results of the three weighted products as output Yin et al. (2017), Dey and Salem (2017).

#### 2.3.4 Performance evaluation

To assess the performance of the prediction model Browne (2000), six performance metrics are used: accuracy, sensitivity, specificity, precision, F1 score, and Matthew’s correlation coefficient (MCC). The formulas for calculating these metrics are as follows:

$$ACC=\frac{TP+TN}{TP+FP+TN+FN}\times 100\%$$ (3)

$$Sensitivity=\frac{TP}{TP+FN}\times 100\%$$ (4)

$$Specificity=\frac{TN}{TN+FP}\times 100\%$$ (5)

$$Precision=\frac{TP}{TP+FP}\times 100\%$$ (6)

$$F_{1}=\frac{2\times TP}{2\times TP+FN+FP}$$ (7)

$$MCC=\frac{TP\times TN-FP\times FN}{\sqrt{\left(TP+FN\right)\times \left(TN+FP\right)\times \left(TP+FP\right)+\left(TN+FN\right)}}$$ (8)

These metrics are used in protein classification because each provides a unique perspective on assessing the performance of a classification model in different aspects. Accuracy measures the proportion of correctly classified samples out of the total samples and is suitable for balanced datasets. Sensitivity or Recall focuses on the model’s ability to correctly identify positive instances, which is crucial when the aim is to minimize missing any positives. Specificity measures the model’s accuracy in identifying negative instances, ensuring that the model does not falsely label too many negatives as positives. Precision focuses on the proportion of positive predictions, which is suitable for scenarios where high accuracy in predicting positives is required. The F1 score is the harmonic mean of precision and sensitivity, ideal for balancing recall and precision in imbalanced datasets. Lastly, MCC provides a comprehensive metric that considers all four quadrants of the confusion matrix, offering a balanced and robust performance assessment, especially useful in highly imbalanced situations. Together, these metrics help researchers thoroughly evaluate and optimize their classification models.

## 3 Experimental results

### 3.1 Comparing performance with state-of-the-art methods on UniSwiss-Tst

In this section, to further illustrate the effectiveness of the proposed LGC-DBP, we will compare it with state-of-the-art methods. On the UniSwissTst test dataset, we compared our model with TargetDBP, iDNAProt-ES Chowdhury et al. (2017), TargetDBP+, MsDBP Du et al. (2019), RF-SVM Zhang et al. (2022), TPSO-DBP Sikander et al. (2023), and DBPboost. All the methods mentioned in this study utilize UniSwiss-Tr as the training dataset and Uniswiss-test as the independent test set. Our approach demonstrates good precision, accuracy, and performance of the MCC.

Table 1 clearly shows that when using Uniswiss-Tr as a training set, LGC-DBP performs exceptionally well in terms of ACC, F1, and Spe. Specifically, the accuracy reaches 88. 26%, the specificity reaches 88. 52%, and the F1 score reaches 0.878.

**TABLE 1:** Performance comparison between LGC-DBP and state-of-the-art methods on UniSwiss-Tr.

Train set	Method	Acc	Sen	Spe	Pre	MCC	F1
Uniswiss-Tr	TargetDBP	73.10	66.93	79.27	76.35	0.465	0.713
	iDNAProt-ES	77.30	73.75	80.84	79.38	0.547	0.765
	TargetDBP+	85.83	82.41	89.24	88.45	0.718	0.853
	MsDBP	67.19	85.30	49.08	62.62	0.369	0.722
	RF-SVM	84.25	87.66	80.84	82.06	0.687	0.848
	TPSO-DBP	87.01	85.30	88.71	88.31	0.741	0.868
	DBPboost	89.32	89.01	89.37	87.26	0.657	0.758
	LGC-DBP	88.26	85.62	88.52	88.65	0.701	0.878

**FIGURE 3:**
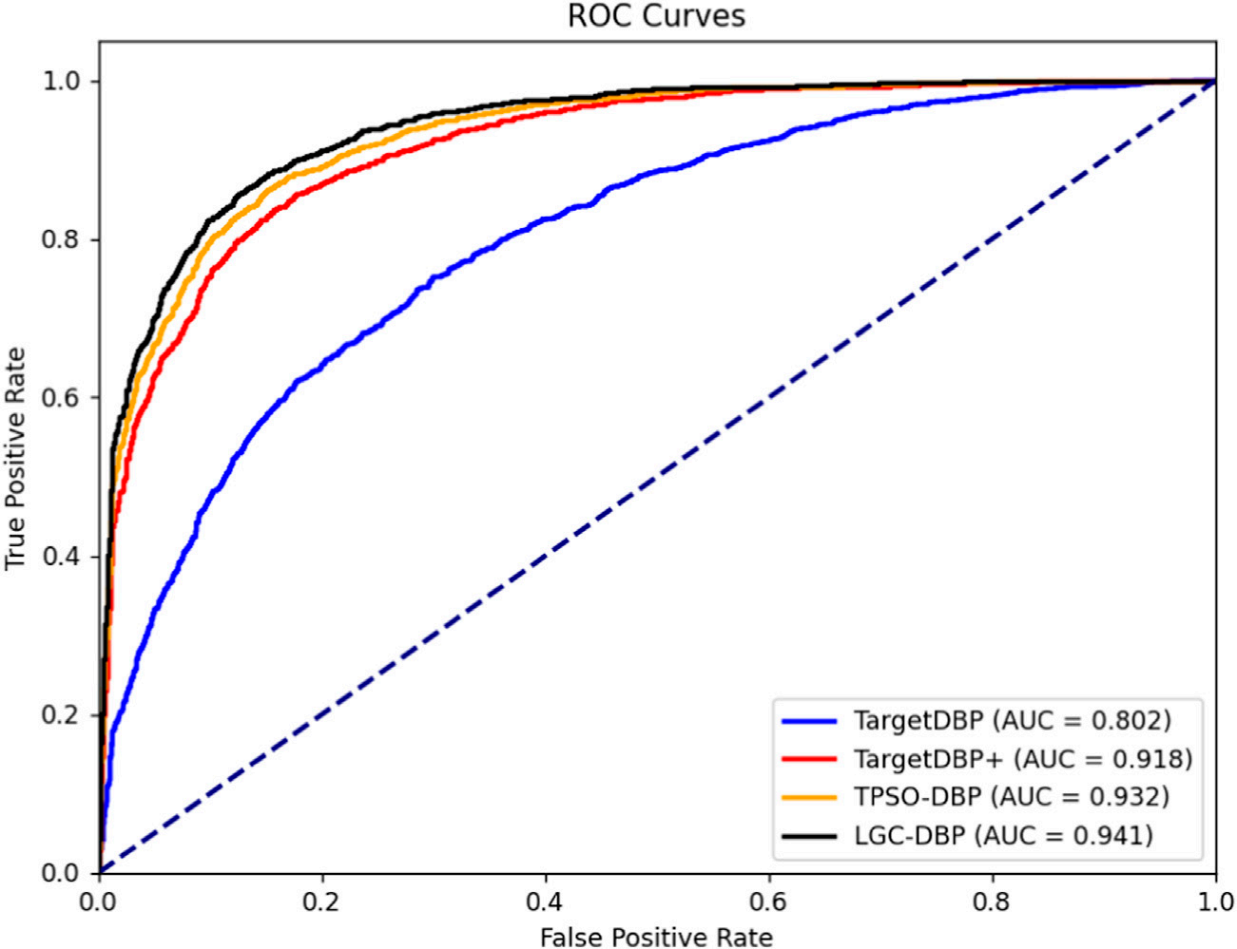
ROC curve comparison.

To further validate the effectiveness of the proposed TPSODBP, we plotted the ROC curves Zweig and Campbell (1993) for TargetDBP, TargetDBP+, TPSO-DBP, and LGC-DBP in the Uniswiss-Test data set, as shown in Figure 3. According to the ROC curves, it is evident that the AUC value for LGC-DBP is higher than those of TargetDBP, TargetDBP+, and TPSO-DBP. The AUC value for LGC-DBP is 0.941, marking increases of 17.33%, 2.51%, and 0.97% over the AUC values for TargetDBP (0.802), TargetDBP+ (0.918), and TPSO-DBP (0.932), respectively.

**FIGURE 4:**
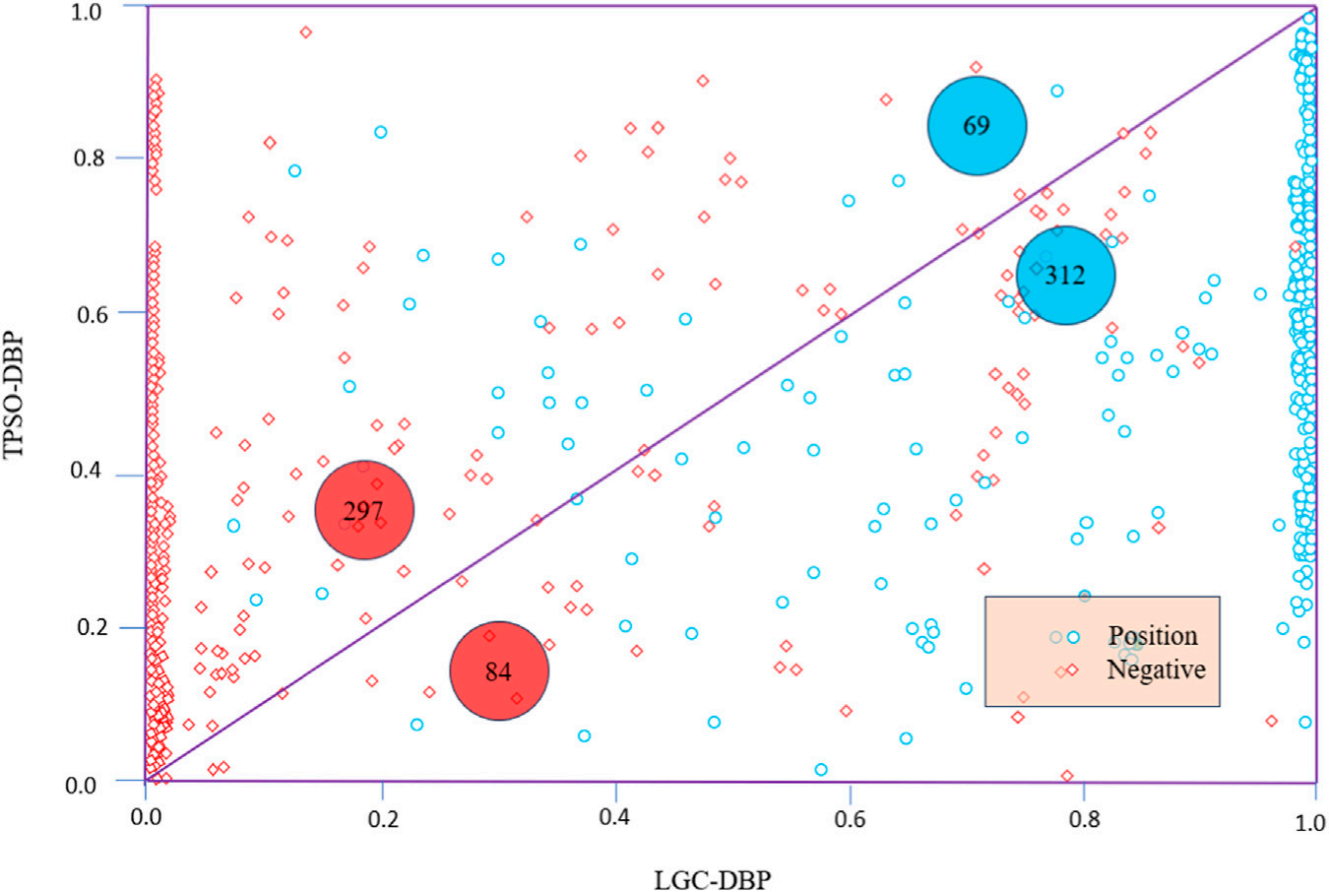
Scatter diagrams of TargetDBP+ and LGC-DBP on UniSwiss-Tst. Blue circles represent positive samples, and red diamonds represent negative samples. The axes represent the probabilities of being predicted as a positive sample. The diagram’s red (or blue) numbers indicate the number of negative (or positive) samples in the upper or lower triangle.

In addition to the ROC curves, Figure 4 shows scatter diagrams for TargetDBP+ and LGC-DBP. LGC-DBP outperforms TargetDBP+ with both negative and positive samples. Specifically, compared to TargetDBP+, LGC-DBP shows better-predicted probability values for 297 negative and 312 positive samples.

### 3.2 Comparing the generalization performance of LGC-DBP with other methods

Generalization Brennan (1992) refers to the ability of a machine learning model to perform well on unseen data. A model that generalizes well can make accurate predictions on the data and new data on which it was trained. This is an essential metric for evaluating the model’s performance because it reflects its adaptability to real-world conditions. A generalizable model is better equipped to handle new situations and data, enhancing its practicality and reliability. In machine learning tasks, especially in real-world applications, the generalization quality often determines the actual effectiveness of the model. Therefore, ensuring a model’s good generalization is crucial to building reliable machine learning systems.

To test the generalization of LGC-DBP, the prediction model was separately trained on the training data sets of other methods (PDB424, PDB1075, and PDB2104), using parameters tuned in UniSwiss-Tr. This training aimed to identify proteins in the Uniswiss-Test dataset. It is important to note that PDB424 serves as the training dataset for DNA-prot; PDB1075 for PSFM-DBT, PseDNA-Pro, Local-DPP, iDNAProPseAAC, HMMBinder, DPP-PseAAC, and DNA-propolis; and PDB2104 for TargetDBP. Further details are provided in Table 2.

**TABLE 2:** Generalization performance comparison between LGC-DBP and state-of-the-art methods on PDB424, PDB1075,PDB2104.

Train set	Method	Acc	Sen	Spe	Pre	MCC	**F**_1_
PDB424	iDNA-Prot	50.06	48.56	51.97	50.27	0.005	0.494
	TargetDBP+	59.38	83.25	34.91	56.41	0.220	0.676
	TPSO-DBP	63.11	52.75	76.06	67.91	0.285	0.594
	LGC-DBP	65.51	51.35	76.28	65.95	0.332	0.591
PDB1075	PSFM-DBT	67.18	87.30	48.35	61.52	0.385	0.722
	PseDNA-Pro	58.74	74.28	41.21	55.82	0.164	0.637
	Local-DPP	52.37	13.53	92.89	66.38	0.106	0.224
	iDNAPro-PseAAC	49.55	64.55	32.63	48.80	−0.030	0.556
	HMMBinder	50.05	99.74	2.36	50.53	0.092	0.671
	DPP-PseAAC	50.25	54.59	55.91	55.32	0.120	0.620
	iDNA-Prot l dis	51.64	72.44	38.85	54.22	0.120	0.620
	TargetDBP+	69.82	66.40	73.23	71.27	0.397	0.688
	TPSO-DBP	70.21	70.08	70.34	70.26	0.404	0.702
	LGC-DBP	70.95	71.13	70.95	70.53	0.398	0.705
PDB2104	TargetDBP	73.10	66.93	79.27	73.10	0.465	0.713
	TargetDBP+	74.28	73.75	74.80	74.28	0.486	0.741
	TPSO-DBP	72.31	79.52	65.09	72.31	0.470	0.742
	LGC-DBP	74.52	73.13	70.58	72.19	0.473	0.745

It can be observed that LGC-DBP demonstrates excellent performance across three different datasets, further proving its strong generalization capability. The consistent performance of the model across diverse datasets indicates its adaptability and robustness to varying data characteristics and distributions. This outstanding generalization performance can be attributed to carefully considering diverse data features in the design of LGC-DBP and the effective optimization strategies employed during model training. Such robust generalization enables LGC-DBP to excel in specific datasets and maintain stable performance when confronted with new and unknown data, providing a reliable solution for DNA-binding protein classification and recognition tasks in practical applications.

Figure 4 displays scatter plots of TargetDBP+ and LGC-DBP alongside ROC curves. LGC-DBP exhibits performance superior to that of TargetDBP+ in negative and positive samples. Specifically, compared to TargetDBP+, LGC-DBP demonstrates better-predicted probability values for 297 negative and 312 positive samples.

### 3.3 Ablation experiments based on LGC-DBP

Ablation experiments aim to assess the impact of individual components or modules on the overall performance of a model. By systematically removing or modifying specific parts of the model and observing changes in performance metrics, researchers can understand the contribution of each component to the model’s effectiveness. These experiments help elucidate the model’s robustness, identify critical features, and optimize its architecture for improved performance. In the context of LGC-DBP, the ablation experiments described in this section involve removing modules such as the channel attention mechanism, LSTM module, or specific layers in the Gated Inception convolution to evaluate their respective contributions to the performance of the DNA-binding protein prediction model. The results of these ablation experiments are shown in Table 3.

**TABLE 3:** Ablation experiments based on LGC-DBP.

LSTM	Gated inception Conv	Improved channel attention	Acc	MCC	F1
	*✓*		76.98	0.673	0.745
*✓*			76.31	0.608	0.699
		*✓*	77.01	0.614	0.715
*✓*	*✓*		77.19	0.669	0.721
*✓*		*✓*	84.00	0.679	0.834
	*✓*	*✓*	86.01	0.691	0.825
*✓*	*✓*	*✓*	88.26	0.701	0.878

From Table 3, it is evident that initially, using a simple, unoptimized LSTM for DNA-binding protein identification results in a decrease in accuracy by 11.95% compared to the complete LGC-DBP model. When considering only one of the components in the model, the LSTM, Gated Inception Conv, or Improved Channel Attention, it is observed that Improved Channel Attention demonstrates the best identification performance. This is attributed to its more rational weighting mechanism and the optimization of channel attention, which align better with the crucial features of DNA-binding proteins. In particular, the most significant decrease in overall model performance is observed when the improved channel attention is removed, thus confirming the previous observation. The second most influential component is the Gated Inception Conv, which highlights its importance. Although the LSTM contributes relatively less to the model, its ability to capture long-term dependencies in protein sequences remains valuable.

### 3.4 The comparative experiments for gated Inception Conv and improved channel attention

**FIGURE 5:**
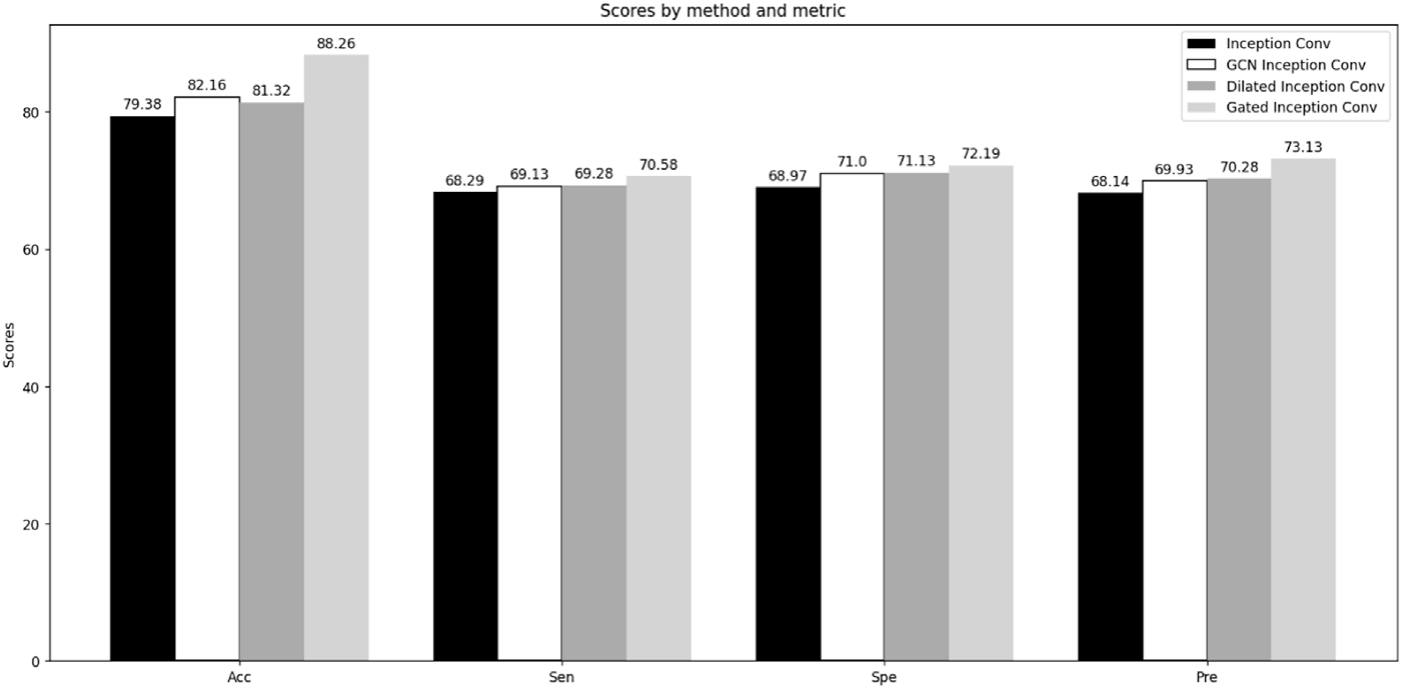
Comparative experiments of Gated Inception Convolution.

The LGC-DBP model comprises three modules: LSTM, Gated Inception Convolution, and Improved Channel Attention. We have made enhancements to the latter two modules. We conducted two sets of comparative experiments to test the effectiveness of our modifications to the Gated Inception Convolution and Improved Channel Attention. The first set of experiments focused on Gated Inception Convolution, where we tested the performance variations of the Inception Conv (unmodified module), GCN Inception Conv, Dilated Inception Conv, and Gated Inception Conv under unchanged conditions. The second set of experiments targeted Improved Channel Attention, where we evaluated the performance changes before and after modifying Channel Attention, also under unchanged conditions. Accuracy, sensitivity, specificity, and precision were selected as metrics to assess whether the modifications to Gated Inception Convolution and Improved Channel Attention improved the overall model performance. The results of the two sets of comparative experiments are shown in Figure 4 and Figure 5.

Through comparative experiments, it can be observed that the performance of the improved Gated Inception Convolution is significantly better than that of the original Inception Convolution. The incorporation of GCN and dilated convolutions has resulted in performance improvements of 2.78% and 1.94%, respectively, for the entire model. This improvement is due to GCN’s suitability for handling data with complex topological structures, such as PSSM matrices. At the same time, dilated convolutions expand the receptive field, aiding in capturing global features of DNA-binding proteins. Furthermore, the weighted function of the gating units enhances the interaction between GCN and dilated convolutions, leading to improved performance. The modified channel attention mechanism also performs better regarding ACC, Spe, Sen, and Pre than the ordinary mechanism. This enhancement is attributed to multiple inputs obtained from different convolutional layers and the weighted mechanism at the excitation level, enabling the channel attention mechanism to identify DNA-binding proteins better.

**FIGURE 6:**
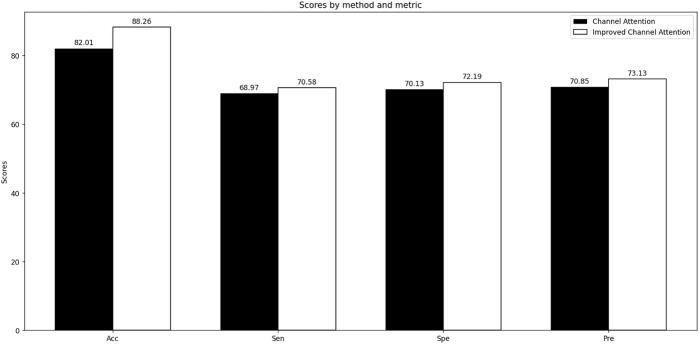
Comparative experiments of Improved Channel Attention.

### 3.5 Case study

A case study selects a sample or dataset from actual data to test the performance of models, algorithms, or techniques. Conducting a case study helps evaluate the effectiveness of models on accurate data, testing their generalization ability and applicability. Through the analysis of case data, one can identify the strengths, weaknesses, and areas for improvement in the model, providing practical insights for solving specific problems. Case studies typically involve data preprocessing, feature engineering, model training, and evaluation, offering comprehensive insights into the model’s real-world application and enabling corresponding improvements and optimizations. In this study, five random samples were selected from Uniswiss-Tst for the case study, and the results are shown in Table 4.

**TABLE 4:** Case Study of LGC-DBP.

	O43058	P32607	Q872F4	Q54C37	F4JGZ1
TargetDBP+	×	×	×	*✓*	×
DBPboost	×	×	*✓*	×	×
LGC-DBP	*✓*	*✓*	*✓*	*✓*	*✓*

From Table 4, we can observe that LGC-DBP successfully predicted these five proteins.The failure of other models in predicting certain protein samples can be attributed to several key limitations, including inadequate feature extraction, structural constraints of the models, insufficient generalization capabilities, and the inability to handle complex data, as well as optimization and parameter tuning issues. Particularly when dealing with proteins with complex structures or specific functional regions, these models fail to effectively capture complex patterns within the sequences or properly manage long-term dependencies between sequences. In contrast, the LGC-DBP model, by integrating advanced deep learning technologies and architectures, has significantly enhanced the comprehensiveness of feature extraction and the generalization ability of the model, thereby demonstrating exceptional performance across a broad range of samples. The LGC-DBP model excels in protein classification, especially in identifying DNA-binding proteins, due to its unique composite architecture, including LSTM, improved Gated Inception Convolution, and an enhanced Channel Attention mechanism. The LSTM component excels at capturing long-term dependencies in sequence data, which is crucial to accurately understanding the order and context of protein sequences. The improved Gated Inception Convolution delves deeper into capturing spatial features and relationships within the input data, thus boosting the model’s capability to handle complex data and enhancing its classification performance. Furthermore, the enhanced Channel Attention mechanism adaptively highlights important features, significantly improving the model’s precision and efficiency. This advanced integrated network structure provides LGC-DBP with a distinct advantage in handling the complexity and diversity of protein sequences, leading to higher precision and superior performance in tasks involving the prediction of DNA-binding proteins.

## 4 Discussion

Studying DNA-binding proteins is crucial for deepening our understanding of gene regulation, cellular functions, and disease mechanisms. Using deep learning (Wang et al., 2023b) to classify DNA-binding proteins enhances our understanding of protein functions and interactions and offers efficient, accurate, and automated analytical advantages. Despite various protein classification methods, these approaches are constrained by limitations such as being confined to known protein structures and functions, lacking completeness, and struggling to capture dynamic changes.

Given these limitations of existing models, we developed a novel protein classification model named LGC-DBP. The LGC-DBP model significantly improves the accuracy of DNA-binding protein classification through several vital enhancements. Firstly, we improved the Inception Convolution module by incorporating GCN and dilated convolutions to replace the original convolutional layers. This enhancement enhances the model’s data representation and classification performance, reducing model complexity and training time. Secondly, we optimized the attention mechanism by integrating inputs from three different scales of convolutional layers and applying sigmoid weighting to these inputs in the fully connected layer. This enhances the model’s ability to recognize complex patterns, improving its performance and generalization across multiple tasks and datasets. Experimental results demonstrate that LGC-DBP outperforms most existing models on numerous datasets, validating the rationale and practicality of the improvements made to various components of the model.

The multi-module fusion strategy employed by LGC-DBP significantly enhances its ability to capture the spatial and temporal characteristics of protein sequences, thereby improving classification accuracy and model generalization. This comprehensive approach makes the model more efficient in handling long sequences and complex protein structures. However, the high complexity of the model increases computational costs, potentially requiring more computational resources and time, especially when dealing with large-scale datasets. We are optimizing algorithms and adopting efficient hardware acceleration techniques to address this issue. Additionally, the complexity of model tuning and maintenance has also increased. So, we plan to use automated machine learning techniques such as automatic hyperparameter optimization to help reduce the burden of manual parameter tuning and further enhance model performance. Despite its limitations, LGC-DBP has achieved promising results in predicting DNA-binding proteins and represents a reliable choice for protein prediction experiments.

## 5 Conclusion

Identifying DBPs is crucial for uncovering information about DNA-protein interactions, significantly contributing to our understanding of biological processes. This paper proposes an LGC-DBP method for identifying DNA-binding proteins to enhance DBP recognition performance. In LGC-DBP, three modules are sequentially connected: LSTM, Gated Inception Convolution, which incorporates gating units for feature modulation, and Improved Channel Attention. Experimental results demonstrate that the LGC-DBP prediction method outperforms most state-of-the-art DBP prediction methods. While LGC-DBP achieves favorable predictive performance, several aspects require further improvement in future research: 1) Enhancing the quality of DBP datasets to better suit model training, 2) Exploring connections between DNA-binding proteins and other protein data to discover novel identification methods, and 3) Continuously refining network architectures to address overfitting issues as training batch sizes increase.

## Data Availability

The data presented in the study are deposited in the github repository, accession url https://github.com/jun-csbio/TargetDBPplus/.
